# Assessing the Risks of Twin Pregnancies

**DOI:** 10.1371/journal.pmed.0020195

**Published:** 2005-06-28

As mothers get older and assisted conception becomes more common in developed countries, the incidence of multiple births—primarily of nonidentical siblings, but also of identical ones—has dramatically increased. Multiple pregnancies are high-risk pregnancies, with preterm delivery and monochorionicity (shared placenta) the major problems. Consequently, efforts are underway to optimize the management of these pregnancies.

While identical (monozygotic) twins are much less common than dizygotic ones, monozygotic twinning events are increased after induced ovulation and in vitro fertilization. Monozygotic twins can be diamniotic dichorionic (two amniotic sacs, two placentas), monoamniotic monochorionic (one amniotic sac, one placenta), or diamniotic monochorionic (two amniotic sacs, one placenta). The last type accounts for approximately two-thirds of all monozygotic twins.

Monochorionic twins are at higher risk because they share a common placenta; they are primarily at risk from circulation abnormalities like twin–twin transfusion syndrome (the smaller twin [donor] does not get enough blood while the larger twin [recipient] becomes volume overloaded) and intrauterine growth restriction. However, the majority of diamniotic monochorionic twin pregnancies do not develop such complications.

Nicholas Fisk and colleagues have studied records of 151 seemingly uncomplicated diamniotic monochorionic pregnancies and found a surprisingly high rate of fetal death: ten unexpected intrauterine deaths occurred in seven of the 151 pregnancies with no prior signs of complications. All deaths occurred within two weeks of a normal scan, at a median gestational age of 34 weeks and 1 day.

The authors conclude that “despite intensive fetal surveillance, structurally normal monochorionic diamniotic twin pregnancies without twin–twin transfusion syndrome and intrauterine growth restriction are complicated by a high rate of intrauterine death.” As the deaths occurred predominantly after 32 weeks' gestation, the authors suggest that the prospective risk for fetal death in these pregnancies might be eliminated by elective preterm delivery after 32 weeks.

In an accompanying Perspective (DOI: 10.1371/journal.pmed.0020180), Jane Cleary-Goldman and Mary D'Alton agree that, despite the limitations of the study (its retrospective nature, small numbers, and lack of dichorionic controls), it highlights a critical question for obstetricians, namely, “when is the ideal gestational age to deliver apparently uncomplicated monochorionic twins?” As Cleary-Goldman and D'Alton discuss, at 32 weeks of gestation many of the risks associated with prematurity have abated, but the remaining ones are not negligible. Until larger prospective observational studies have been conducted, balancing these risks remains challenging.

